# Feline SCCs of the Head and Neck Display Partial Epithelial-Mesenchymal Transition and Harbor Stem Cell-like Cancer Cells

**DOI:** 10.3390/pathogens12111288

**Published:** 2023-10-27

**Authors:** Stefan Kummer, Andrea Klang, Carina Strohmayer, Ingrid Walter, Christoph Jindra, Sibylle Kneissl, Sabine Brandt

**Affiliations:** 1 VetCore Facility for Research, University of Veterinary Medicine, 1210 Vienna, Austria; stefan.kummer@vetmeduni.ac.at (S.K.); ingrid.walter@vetmeduni.ac.at (I.W.); 2 Institute of Pathology, Department of Pathobiology, University of Veterinary Medicine, 1210 Vienna, Austria; andrea.klang@vetmeduni.ac.at; 3 Clinical Unit of Diagnostic Imaging, Department for Companion Animals and Horses, University of Veterinary Medicine, 1210 Vienna, Austria; carina.strohmayer@vetmeduni.ac.at (C.S.); sibylle.kneissl@vetmeduni.ac.at (S.K.); 4 Institute of Morphology, Department of Pathobiology, University of Veterinary Medicine, 1210 Vienna, Austria; 5 Research Group Oncology (RGO), Clinical Unit of Equine Surgery, Department for Companion Animals and Horses, University of Veterinary Medicine, 1210 Vienna, Austria; christoph.jindra@vetmeduni.ac.at; 6 Division of Molecular Oncology and Hematology, Karl Landsteiner University of Health Sciences, 3500 Krems an der Donau, Austria

**Keywords:** HNSCC, cats, partial epithelial-mesenchymal transition, cancer stem cells, IHC, IF

## Abstract

Squamous cell carcinoma of the head and neck (HNSCC) is a malignant cancer disease in humans and animals. There is ample evidence that the high plasticity of cancer cells, i.e., their ability to switch from an epithelial to a mesenchymal, endothelial, and stem cell-like phenotype, chiefly contributes to progression, metastasis, and multidrug resistance of human HNSCCs. In feline HNSCC, the field of cancer cell plasticity is still unexplored. In this study, fourteen feline HNSCCs with a known feline papillomavirus (FPV) infection status were subjected to histopathological grading and subsequent screening for expression of epithelial, mesenchymal, and stem cell markers by immunohistochemistry (IHC) and immunofluorescence staining (IF). Irrespective of the FPV infection status, all tumors except one corresponded to high-grade, invasive lesions and concurrently expressed epithelial (keratins, E-cadherin, β-catenin) and mesenchymal (vimentin, N-cadherin, CD146) proteins. This finding is indicative for partial epithelial-mesenchymal transition (pEMT) events in the lesions, as similarly described for human HNSCCs. IF double staining revealed the presence of CD44/CD271 double-positive cells notably within the tumors’ invasive fronts that likely correspond to cancer stem cells. Taken together, the obtained findings suggest that feline HNSCCs closely resemble their human counterparts with respect to tumor cell plasticity.

## 1. Introduction

**Figure 1:**
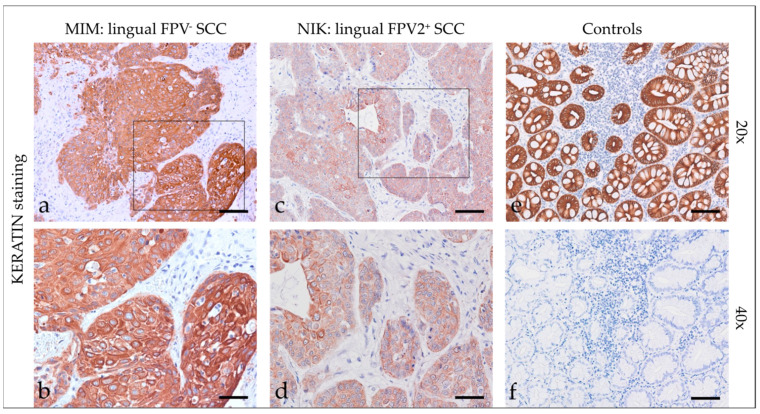
Keratin staining of feline SCC sections and controls. The figure depicts two representative KRT-stained feline HNSCC sections and controls. Framed areas in (**a**,**c**) are presented at higher magnification (40×; (**b**,**d**)). The lingual, FPV-negative HNSCC of cat MIM displayed intense diffuse KRT staining of tumor cells at 20× (**a**) and 40× magnification (**b**). The FPV2 positive lingual SCC of cat NIK revealed mild to moderate diffuse KRT staining of tumor cells at 20× (**c**) and 40× magnification (**d**). (**e**) Positive control, KRT staining of feline colon. (**f**) Negative control, mock staining (no-primary Ab) of feline colon. Scale bar = 80 µm.

Squamous cell carcinoma (SCC) is a common, potentially metastasizing tumor disease arising from cutaneous or mucosal keratinocytes. In humans, a considerable proportion of SCCs are caused by infections with high-risk human papillomaviruses (hrHPVs) [1]. In cats, SCCs mainly affect the head and neck, i.e., the oronasal (Figure 1), pharyngeal, laryngeal, and periocular regions, as well as the aural pinnae [2]. Although feline head and neck SCCs (HNSCCs) are associated with high mortality rates, the etiology of the disease remains largely unclear. In a critical review addressing this unresolved issue, infection by feline papillomaviruses (FPVs) was identified as potential (co-)factor driving feline oral SCC (FOSCC) development [3]. This finding is corroborated by the recently reported detection of FPV types 1-5 in a subset of FOSCCs [4]. Other factors such as overexposure to UV radiation (nasal planum, aural pinnae), oral comorbidities, canned food, or exposure to tobacco smoke are likewise suspected to promote HNSCC onset and progression in cats [2,3]. Treatment of human, and to some extent, feline HNSCCs is mainly based on surgery, chemo-, radio-, and immunotherapy, or combinations of these therapeutic approaches. However, although recent developments and approvals of innovative tumor therapeutics have significantly improved cancer management, HNSCC therapy continues to be challenging or futile in human and animal patients including cats [2,5].

In humans, there is ample evidence that malignant progression and therapy resistance of HNSCCs is chiefly due to the high phenotypic plasticity of tumor cells. Following a cellular program termed epithelial-mesenchymal transition (EMT), epithelial tumor cells can reversibly switch to a mesenchymal phenotype via E-cadherin suppression allowing EMT transcription factors to promote enhanced expression of mesenchymal proteins. The latter drives extracellular matrix (ECM) degradation, cell motility, invasion, and metastasis [5,6,7,8,9]. In addition, tumor cells can adopt an endothelial phenotype that can build up an alternative system of microvessels (“vasculogenic mimicry”) facilitating metastases [10]. Moreover, tumor cells can acquire a stem cell-like phenotype that has the ability to self-renew, transdifferentiate, and migrate [5,11,12,13,14]. Long-lived cancer stem cells (CSCs) show a pronounced resistance to stress factors such as DNA damage, reactive oxygen species, or hypoxia, and because of this, they are multidrug resistant. Their crucial involvement in tumor progression and reoccurrence, immune evasion, and metastasis has been unequivocally demonstrated [5,11,14].

In vitro, human cancer cells can undergo complete EMT, as reflected by a total loss of epithelial and the full acquisition of mesenchymal properties. In vivo, however, cancer cells rather adopt a hybrid epithelial/mesenchymal (E/M) phenotype characterized by concurrent expression of epithelial and mesenchymal proteins [15,16,17]. This ability of cancer cells to undergo partial EMT (pEMT) is associated with a higher metastatic potential. Circulating tumor cells (CTCs) exhibit a more aggressive, therapy-resistant pEMT phenotype that exits the blood stream and establishes metastases more effectively [18,19]. In addition, pEMT endows cancer cells with the ability to migrate collectively as multicellular clusters [16].

In cats, information on the role of EMT and CSCs in HNSCC development, progression, and metastasis is poor. In 2012, Pang and colleagues reported on gefitinib-induced EMT and the presence of putative CSCs in cultured feline SCC cells [20]. More recently, Harris et al. provided evidence of EMT via molecular biological and immunohistochemical analysis of ten FOSCC biopsies and three FOSCC cell lines [21]. To our knowledge, no other works in this field have been published so far. This paucity of knowledge prompted us to grade a set of feline papillomavirus (FPV)-positive and -negative HNSCCs, and then comparatively screen the respective tumor sections for expression of selected epithelial, mesenchymal, endothelial, and stem cell markers using single immunohistochemical and double immunofluorescence staining. We provide robust evidence of pEMT occurring in all analyzed tumors and of the intratumoral presence of cell subsets that most likely represent CSCs. No significant correlation between the presence and extent of EMT/CSCs and the FPV infection status was noted.

## 2. Materials and Methods

### 2.1. Sample Material

The study was conducted on HNSCC tissue originating from 14 feline patients (Table 1). Tumor material was collected at the Veterinary University of Vienna, Austria, during therapeutic surgical excision or requested necropsy with the owners’ written consent. Patient and tumor specifications are provided in Table 1. These also include the feline papillomavirus (FPV) infection status that was determined by FPV type-specific PCR from HNSCC DNA in a previous study [4].

### 2.2. Tumor Grading

FFPE tumor sections were routinely stained with hematoxylin and eosin (HE) [22], and histopathologically analyzed using an Olympus BX45 light microscope. Staging was carried out by pathologist A.K. according to the malignancy grading system of oral SCC proposed by Anneroth and colleagues [23]. This system is based on the cumulative scoring of the degrees of tumor keratinization, nuclear polymorphisms, mitotic cells, invasion patterns and stages, and lymphoplasmocytic infiltration, leading to total scores ranging between 5 and >20, with 5–10 points corresponding to grade 1, 11–15 points to grade 2, 16–20 points to grade 3, and >20 points to grade 4 [23].

### 2.3. Immunohistochemical Staining (IHC)

FFPE tumor sections (2.5 μm) were assessed by a single labeling approach for expression of keratins (KRT), β-catenin, Vimentin, CD146, COX-2, CD271 (p75NTR), and CD44. To this end, sections were deparaffinized with xylene, and gradually rehydrated in 100%, 96%, and 70% ethanol. Then, sections were treated with 0.3% H_2_O_2_/methanol to block peroxidase activity. Heat-induced epitope retrieval (HIER) was performed in 0.01 M citrate buffer (pH6) or Tris-EDTA buffer (pH 9) for 30 min in a steamer at 94–100 °C. To minimize unspecific bindings, sections were blocked with 1.5% normal goat serum. Incubation with the primary antibody (Ab; for Abs and staining specifications see Supplementary Table S1) was conducted at 4 °C overnight. After washing with phosphate-buffered saline (PBS), sections were incubated with secondary horseradish peroxidase (HRP)-conjugated Ab for 30 min at room temperature. Ab-bound protein was visualized with diaminobenzidine 123 (DAB) chromogen (Richard Allan Scientific, Kalamazoo, MI, USA). HE was used for nuclear counterstaining. Evaluation of signals was semiquantitative and performed by blinded investigator AK using an Olympus BX45 light microscope. The following staining characteristics were assessed: (i) intracytoplasmic versus membranous or nuclear immunostaining; (ii) DAB signaling intensity that was classified as absent, mild, moderate, or strong; (iii) the labeling pattern, i.e., diffuse labeling of all tumor cell layers, patchy labeling with irregular distribution, and special labeling patterns in the center of tumor islets or the invasive front; (iv) estimated percentage of positive tumor cells (<10%, <50%, >50%, 100%). 

For evaluation, five highly representative fields were selected at 100× magnification. Images were captured using an Olympus BX51 microscope equipped with an Olympus camera UC90 (Olympus, Vienna, Austria). High-resolution images were captured using a Zeiss LSM880 Airyscan confocal microscope (Carl Zeiss AG, Jena, Germany). All antibody and further staining specifications are provided in detail in Supplementary Table S1).

### 2.4. Immunofluorescent Staining (IF)

In an IF double staining approach, selected FFPE sections (2.5 µm) of FPV-positive and FPV-negative HNSCCs were deparaffinized with xylene and gradually rehydrated in 100%, 96%, and 70% ethanol. Pretreatment was performed with TRIS-EDTA-buffer (pH9) for 30 min in a steamer at 94–100 °C for epitope demasking. Following blocking of unspecific binding sites (Fc-receptors) with 1.5% normal goat serum, sections were incubated with a mixture of primary anti-KRT and anti-vimentin, or anti-CD44 and anti-CD271 Abs at 4 °C overnight. After washing, sections were incubated with a mixture of secondary, Alexa Fluor 488- and 568-conjugated Abs for 60 min. For E-cadherin/N-cadherin double staining, tyramide signal amplification was carried out to reach a better signal-to-noise ratio. After antigen retrieval with Tris-EDTA-buffer (pH9) for 30 min in the steamer, and quenching of endogenous peroxidase with 3% hydrogen peroxide, sections were incubated with the first primary antibody against N-cadherin at 4 °C overnight. After incubation of the secondary anti-mouse antibody for 60 min, the signal was developed with an Alexa 488-labeled tyramide solution for 10 min. After elution of the antibody with 0.01 M citrate buffer (pH6), boiling for 15 min in the microwave, and blocking of unspecific Fc-receptors, the sections were incubated with the second primary anti-E-cadherin antibody at 4 °C overnight. Then, binding of this second primary antibody was detected by incubation with the secondary anti-mouse antibody and subsequent development with Alexa 568-labeled tyramide solution. Finally, nuclei were counterstained with DAPI (Sigma Aldrich, St. Louis, MO, USA), and sections were mounted with Aqua-PolyMount (Polysciences, Szabo-Scandic, Vienna, Austria). Finally, all sections were digitized using a slide scanner (3DHistech, Budapest, Hungary) equipped with a 20× lens. All antibody and further staining specifications are provided in detail in Supplementary Table S1.

### 2.5. Statistics

The significance of differences regarding the presence versus absence of intratumoral FPV infection in relation to selected tumor characteristics was determined by a chi-square test for goodness-of-fit (https://www.socscistatistics.com/tests/goodnessoffit/default2.aspx; accessed on 1 March 2023).

## 3. Results

### 3.1. All but One Feline HNSCCs Corresponded to Invasive Lesions

The 14 feline HNSCCs were graded according to the protocol proposed by Anneroth and colleagues [23]. Overall, all lesions except one (CAL; grade 2) corresponded to SCCs of grade 3 or 4 (Table 2). FPV-positive and FPV-negative tumors did not significantly differ with respect to keratinization, nuclear polymorphism, mitotic cells, invasiveness, or lymphoplasmacytic infiltration. 

### 3.2. Immunohistochemical Staining Results

#### 3.2.1. Keratins

Staining of feline HNSCCs was conducted using primary pan-KRT antibodies recognizing a broad spectrum of low and high molecular weight KRTs (Supplementary Table S1). Feline HNSCCs scored KRT-positive to various extents and with various intensities. KRT expression was consistently confined to the cytoplasm. In sections of two SCCs, KRT staining was more intense in the center of tumorous infiltrates (Figure 1). Detailed KRT staining results are listed in Table 3. The feline colon sections used as the positive control (+c) exhibited intense KRT staining of mucosal epithelial cells. No signal was exhibited by the no-primary Ab control section of feline colon (-c), as anticipated (Figure 1).

#### 3.2.2. β-Catenin

**Figure 2:**
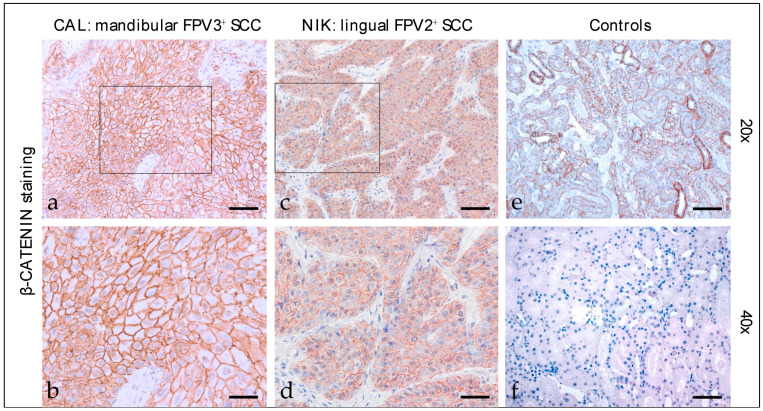
β-catenin staining of feline SCC sections. The figure depicts two representative feline HNSCC sections stained for β-catenin, and controls. Framed areas in (**a**,**c**) are presented at higher magnification (40×; (**b**,**d**)). The mandibular, FPV3-positive SCC of patient CAL exhibited strong, diffuse, predominantly membranous β-catenin expression (**a**,**b**). A diffuse, moderate, membranous β-catenin staining signal was noted in case of the FPV2-positive, lingual SCC of cat NIK (**c**,**d**). (**e**) Positive control, β-catenin-positive tubuloepithelial cells. (**f**) Negative control, mock-stained (no primary Ab) feline kidney section. Scale bar = 80 µm.

Beta-catenin staining of feline HNSCC sections revealed predominantly intensive, diffuse, membranous expression of the molecule in most cases (Figure 2). A patchy signal was evident in one mandibular SCC. A centrally accentuated signal was found in one oral SCC. Blurred cytoplasmic β-catenin expression was only observed occasionally. Detailed β-catenin staining results are listed in Table 3. The feline kidney sections used as the positive control exhibited β-catenin staining, whilst the no-primary Ab control tested negative (Figure 2).

#### 3.2.3. Vimentin

**Figure 3:**
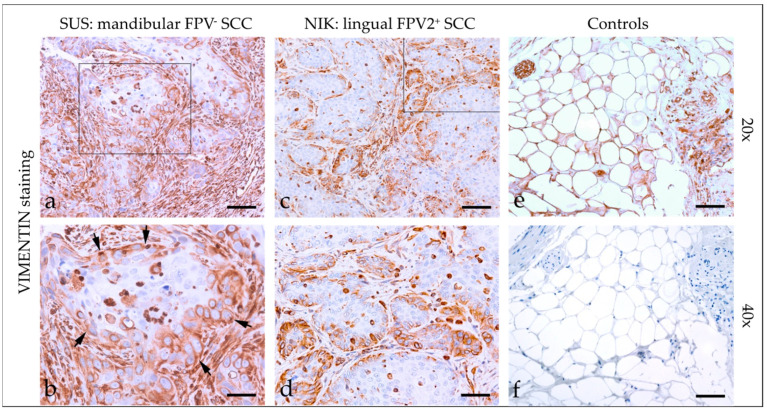
Vimentin staining of feline SCC sections. The figure depicts a representative selection of vimentin staining results. Framed areas in (**a**,**c**) are presented at higher magnification (40×; (**b**,**d**)). (**a**,**b**) Detection of vimentin in >50% of tumor cells in a mandibular, FPV-negative SCC (SUS). Note the immunopositive desmoplastic mesenchymal stroma adjacent to tumorous infiltrates ((**b**) black arrows). (**c**,**d**) Patchy, predominantly marginal distribution of cytoplasmic vimentin staining in < 50% of tumor cells in a lingual, FPV2-positive SCC (NIK). (**e**,**f**) Controls; (**e**) vimentin-positive adipocytes and nerve fibers in feline kidney and (**f**) mock-stained feline kidney control. Scale bar = 80 µm.

Vimentin staining (Figure 3) of feline HNSCC yielded an intensive cytoplasmic signal of tumor cells with patchy distributions in most sections and signals of varying intensity in two cases. Percentages of vimentin-positive cells greatly varied, ranging from <10% to >50%. Detailed vimentin staining results are listed in Table 3. Tissues from feline kidney sections used as the positive control exhibited positive vimentin staining of mesenchymal cells. No signal was exhibited by the no-primary Ab control section (Figure 3).

#### 3.2.4. CD146

**Figure 4:**
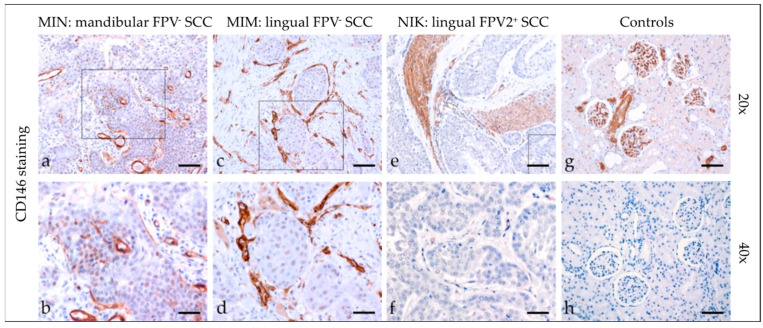
CD146 staining of feline SCC sections. The figure depicts a representative selection of CD146 staining results. Framed areas in (**a**,**c**,**e**) are presented at a higher magnification (40×; (**b**,**d**,**f**)). (**a**,**b**) Mild patchy intracytoplasmic and intranuclear CD146 staining of tumor cells in a mandibular, FPV-negative, mandibular SCC (MIN). (**c**,**d**) Diffuse mild CD146 staining in an FPV-negative, lingual SCC (MIM). (**e**,**f**) Diffuse negative CD146 staining in a FPV2-positive, linguolabial SCC (NIK). Note tumorous infiltrates within arterial vessels. (**g**,**h**) Controls; CD146 (**g**) positive feline kidney sections (**h**), and mock staining feline kidney control. Scale bar = 80 µm.

CD146 (CAM) has a crucial role in cell–cell and cell–matrix adhesion. It was originally identified as a melanoma marker. More recent evidence emphasizes its multifaceted role in various physiological and pathological processes including (tumor) cell migration [24]. Most tumor sections demonstrated diffuse mild cytoplasmic CD146 staining (Figure 4). In two cases, some tumor cells displayed more intensive nuclear expression of the protein. In two further cases, tumor cells scored negative for CD146. Detailed findings of CD146 staining are listed in Table 3. Feline kidney sections exhibited Ab-binding to CD146 antigen, whilst mock-stained sections scored negative (Figure 4).

#### 3.2.5. Cyclooxygenase-2 (COX-2)

**Figure 5:**
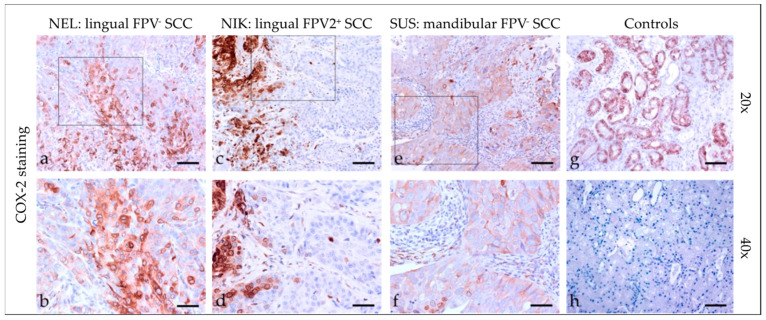
COX-2 staining of feline SCC sections. The figure depicts a representative selection of COX-2 staining results. Framed areas in (**a**,**c**,**e**) are presented at a higher magnification (40×; (**b**,**d**,**f**)). (**a**,**b**) Mild to strong, membranous, and cytoplasmic COX-2 staining of >50% of tumor cells in a lingual, FPV-negative SCC (NEL). (**c**,**d**) <10% COX-2 positive tumor cells at the invasive fronts of tumor islets in a lingual, FPV2-positive SCC (NIK). (**e**,**f**) Mild, diffuse, membranous COX-2 staining in a mandibular, FPV-negative SCC (SUS). (**g**,**h**) Controls; (**g**) COX-2-stained feline kidney tubuloepithelial cells (positive control) and (**h**) no-primary Ab control. Scale bar = 80 µm.

COX-2 staining (Figure 5) of feline SCC sections revealed a cytoplasmic and/or membranous localization of the enzyme, with staining exhibited by <10% to >50% of tumor cells. In one case, COX-2 staining was most pronounced within the tumorous infiltrative fronts. Detailed COX-2 staining results are listed in Table 4. The feline kidney sections that served as the positive controls exhibited COX-2 labeling in tubuloepithelial cells, whereas no signal was obtained when omitting the primary Ab (negative control; Figure 5).

#### 3.2.6. CD44

**Figure 6:**
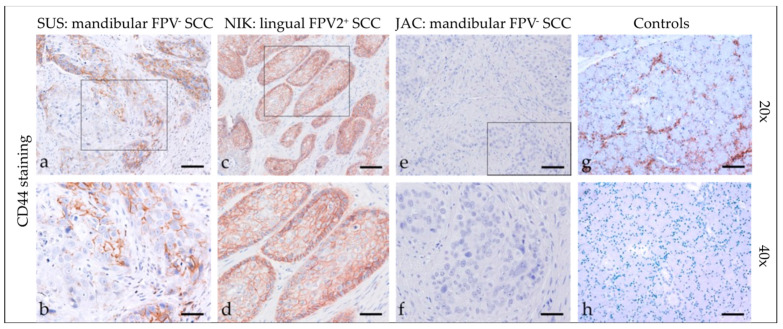
CD44 staining of feline SCC sections. The figure depicts a representative selection of CD44 staining results. Framed areas in (**a**,**c**,**e**) are presented at higher magnification (40×; (**b**,**d**,**f**)). (**a**,**b**) Patchy, membranous CD44 staining of <50% of tumor cells in a mandibular, FPV-negative SCC (SUS). (**c**,**d**) Diffuse, membranous CD44 staining of >50% of tumor cells notably within the margins of a lingual, FPV2-positive SCC (NIK). (**e**,**f**) No CD44-signal in a mandibular, FPV3-positive SCC (JAC). (**g**,**h**) Controls; CD44-positive (**g**) and mock-stained ((**h**) negative control) feline salivary gland epithelial cells. Scale bar = 80 µm.

Feline HNSCCs were assessed for CD44 expression (Figure 6) and revealed predominantly patchy, membranous staining of varying intensity in <10% to >50% of tumor cells. In some cases, the signal was cytoplasmic. In four cases, CD44 staining was more prominent within tumorous infiltrative fronts. Detailed CD44 staining results are listed in Table 4. The feline salivary gland sections used as the positive control exhibited intense CD44 staining of epithelial cells. No signal was exhibited by the no-primary Ab control section (Figure 6).

#### 3.2.7. CD271 (p75NTR)

**Figure 7:**
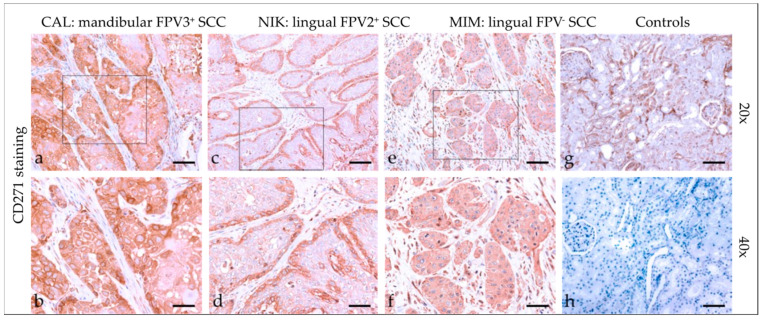
CD271 staining of feline SCC sections. The figure depicts a representative selection of CD271 staining results. Framed areas in (**a**,**c**,**e**) are presented at higher magnification (40×; ((**b**,**d**,**f**)). (**a**,**b**) Diffuse cytoplasmic CD271 staining in a mandibular SCC (CAL). (**c**,**d**) Mild to moderate CD271 staining accentuated within the margins of a lingual, FPV2-positive SCC (NIK). (**e**,**f**) Diffuse, mild CD44 staining in a lingual, FPV-negative SCC (MIM). (**g**,**h**) Controls; CD44-positive (**g**) and mock-stained negative (**h**) feline kidney sections. Scale bar = 80 µm.

Tumor sections demonstrated mild to strong cytoplasmic CD271 staining (Figure 7), with predominantly diffuse distribution. In one case, the signal revealed patchy staining. A marginally accentuated CD271 staining pattern was noted in two cases. Detailed findings of CD271 staining are listed in Table 4. Feline kidney sections exhibited Ab-binding to CD271 antigen, whilst mock-stained kidney sections scored negative (Figure 7).

#### 3.2.8. Feline HNSCCs Scored Positive for EMT and CSC Markers

Target-specific IHC staining results for all tumors, i.e., the staining intensity (I) and distribution (D), and the percentage of positive cells are presented in Table 3 and Table 4.

**Table 3:** Results of IHC staining of feline HNSCC sections for keratin, β-catenin, and vimentin.

Code	Tumor Localization	Keratin			β-Catenin			Vimentin		
		I	D	% +Cells	I	D	% +Cells	I	D	% +Cells
CAL *	mandibular	3	diffuse	100	3	diffuse	100	3	patchy	<10
JAC *	mandibular	2	diffuse	100	3	diffuse	100	3	patchy	<10
MAX *	oromandibular	3	diffuse	100	3	diffuse	100	3	patchy	<50
MIM	lingual	3	diffuse	100	2	diffuse	100	1–3	patchy	>50
MIN	mandibular	1–2	diffuse	100	2	diffuse	100	3	patchy	<50
MMI	mandibular	3	diffuse	100	1	Patchy	>50	3	patchy	<10
NEL	lingual	1–2	diffuse	100	3	diffuse	100	2–3	patchy	<50
NIK *	lingual, labial	1–2	diffuse	100	2	diffuse	100	3	patchy	<50
PAG	oral	3	diffuse	100	3	diffuse	100	3	patchy	<10
PUP	nasopharyngeal, orbital	2–3	diffuse	100	3	diffuse	100	3	patchy	<10
SHA	mandibular	3	diffuse	100	3	diffuse	100	3	patchy	<50
SNO	mandibular	3	diffuse	100	3	diffuse	100	3	patchy	<50
SUR *	nasopharyngeal	2–3	diffuse	100	3	diffuse	100	3	patchy	>50
SUS	mandibular	3	diffuse	100	3	diffuse	100	3	patchy	>50

I—staining intensity; D—staining distribution; % +cells—percentage of positive cells; *: FPV-positive.

**Table 4:** Results of IHC staining of feline HNSCC sections for CD146, COX-2, CD44, and CD271.

Code	CD146			COX-2			CD44			CD271		
	I	D	% +Cells	I	D	% +Cells	I	D	% +Cells	I	D	% +Cells
CAL *	1	diffuse	100	0–3	patchy	<50	0–3	patchy	<50	1–3	diffuse	100
JAC *	1–2	patchy	>50	0–3	patchy	>50	0–2	patchy	<10	2–3	diffuse	100
MAX *	1	diffuse	100	0–2	patchy	<50	0	-	-	3	diffuse	100
MIM	1	diffuse	100	0–3	patchy	<50	0–2	patchy	>50	2–3	diffuse	100
MIN	1–2	diffuse	100	0–2	patchy	<10	0–3	patchy	<50	1–3	diffuse	100
MMI	0	-	0	0–2	patchy	<10	0–1	patchy	<10	2	patchy	<50
NEL	1–2	diffuse	100	1–3	patchy	>50	0–3	patchy	<50	1–2	diffuse	100
NIK *	0	-	0	0–3	patchy	<10	0–3	patchy	>50	1–2	diffuse	100
PAG	1	diffuse	100	0–2	patchy	<10	0–2	patchy	>50	2	diffuse	100
PUP	1	diffuse	100	0–2	patchy	>50	0–3	patchy	<50	1–3	diffuse	100
SHA	1	diffuse	100	0–3	patchy	<50	3	diffuse	100	2–3	diffuse	100
SNO	1	diffuse	100	0–2	patchy	<50	0–3	patchy	<50	2–3	diffuse	100
SUR *	1–2	diffuse	100	0–3	patchy	>50	3	patchy	<50	1–2	diffuse	100
SUS	1	diffuse	100	0–3	patchy	>50	0–3	patchy	<50	1–3	diffuse	100

I—staining intensity; D—staining distribution; % +cells—percentage of positive cells; *: FPV-positive.

### 3.3. IF Double Staining Results Are Suggestive of pEMT and the Presence of CSCs in Feline HNSCCs

#### 3.3.1. Keratin and Vimentin

**Figure 8:**
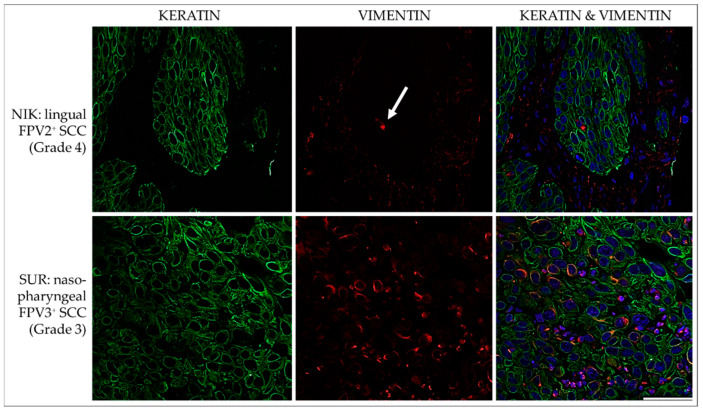
IF double staining for keratins and vimentin. The figure presents confocal images of tumor sections double stained for keratins (green) and vimentin (red). The sections originate from two representative feline HNSCCs, i.e., an FPV2-positive linguo-labial SCC (NIK; top row), and an FPV3-positive nasopharyngeal SCC (SUR; bottom row). Top row: Virtually all tumor cells score positive for expression of keratins, but vimentin staining signals are poor. A single, clearly vimentin-positive cell within the lesion is indicated by a white arrow. Bottom row: Double IF staining reveals co-expression of keratins and vimentin in a subset of tumor cells. Nuclei were visualized by DAPI (blue). Scale bar = 50 µm.

In all but two FPV-negative cases (NEL, MMI), simultaneous intracytoplasmic expression of KRTs and vimentin was evident in varying numbers of cells and at various intensities in FPV-positive and -negative feline HNSCCs. Percentages of double-stained cells varied, ranging from <10% to <50%. The feline pancreas sections used as the positive control exhibited positive vimentin staining of mesenchymal cells and keratin staining of epithelial cells. No signal was exhibited by the no-primary Ab control section. Representative results are depicted in Figure 8.

#### 3.3.2. E-Cadherin and N-Cadherin

**Figure 9:**
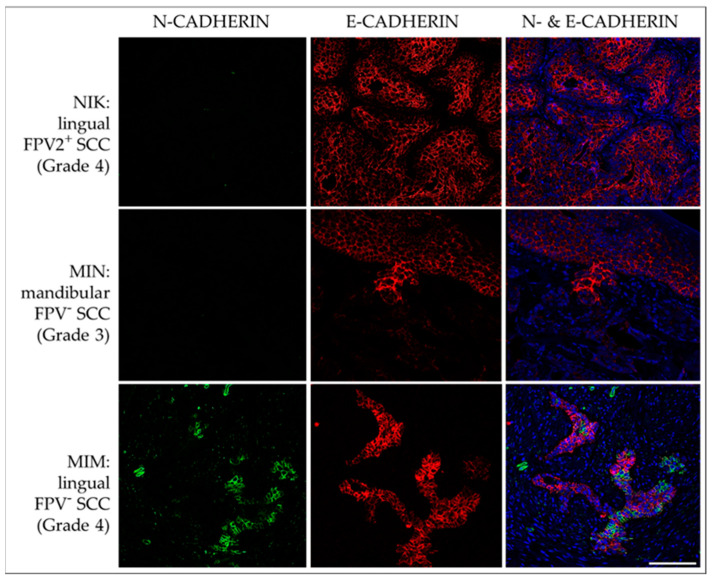
IF double staining of feline HNSCC sections for E-cadherin and N-cadherin. The figure depicts confocal images of E-cadherin (red) and N-cadherin (green) double-stained feline HNSCC sections derived from patients NIK (top row), MIN (middle row), and MIM (bottom row). Top row: An FPV-2-positive, linguolabial, grade-4 SCC with strong E-cadherin expression and negative N-cadherin staining. Middle row: Significant loss of E-cadherin expression within the transitional zone of the tumor, and virtually no N-cadherin-expression in an FPV-negative, mandibular, grade-3 SCC (MIN). Bottom row: Transition from E-cadherin to N-cadherin-expression in a subset of tumor cells in an FPV-negative, lingual, grade-4 SCC (MIM). Nuclei were visualized by DAPI (blue). Scale bar = 100 µm.

The diffuse membranous E-cadherin expression was observed in all FPV-positive and -negative lesions as demonstrated by the variable staining intensities independent of the grade of the tumors’ differentiation. Membranous N-cadherin expression was focally evident in only one FPV-negative (MIM) and one FPV1-positive SCC (JAC). No signal was exhibited by the no-primary Ab control section. Representative results are depicted in Figure 9.

#### 3.3.3. CD44 and CD271

**Figure 10:**
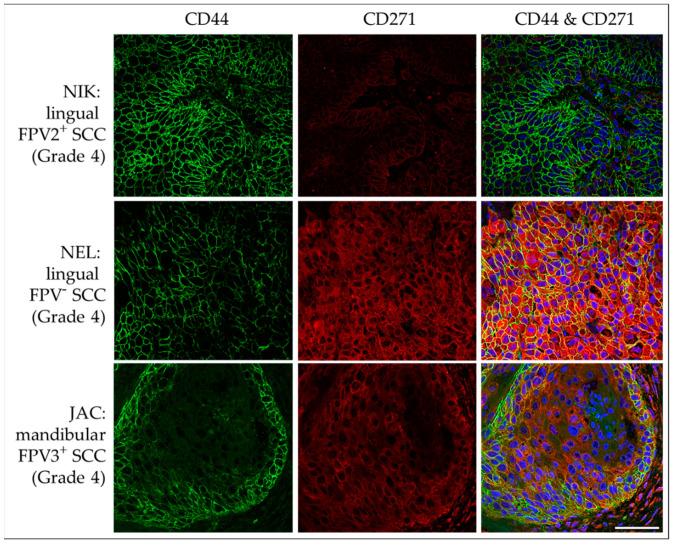
IF double staining of feline HNSCC sections for CD44 and CD271. The figure depicts confocal images of CD44 (green) and CD271 (red) double-stained feline HNSCC sections. These are derived from a FPV2-positive, linguolabial, grade-4 SCC (NIK; top row), an FPV-negative, lingual, grade-4 SCC (NEL; middle row), and an FPV3-positive, mandibular, grade-4 SCC (JAC; bottom row). Top row: Strong, diffuse CD44 expression and a CD271 signal receding into the background are noted. Middle row: Intense CD44-CD271 double staining is observed throughout the section. Bottom row: Mild CD44-CD271 double labeling with accentuated CD44 expression within tumor margins is observed. Nuclei were visualized by DAPI (blue). Scale bar = 50 µm.

IF double staining of HNSCC sections revealed membranous CD44 and cytoplasmic CD271 signal localization with varying intensity in <10% to >50% of tumor cells of FPV-positive and -negative lesions. In many cases, the intensity of the CD44 staining was significantly stronger at the periphery of tumorous infiltrates, i.e., the invasive tumor fronts. No signal was exhibited by the no-primary Ab control section. Representative results are depicted in Figure 10.

## 4. Discussion

The ability of tumor cells to reversibly switch from an epithelial to a mesenchymal, endothelial, or stem cell-like phenotype represents a hallmark of malignant cancer progression and metastasis, all the more since phenotype transformation is associated with multidrug resistance (MDR) [18,25]. In feline oronasal SCC, there is only little information on the plasticity of tumor cells available thus far [20,21]. In 2013, Pang and colleagues reported on the formation of sphere colonies upon starvation of feline SCC cells. These spheres consisted of cells with CSC properties, including the resistance to radiation, chemotherapy, and the EGFR inhibitor gefitinib [20]. In 2013, Harris et al. were the first to provide evidence of EMT ex vivo by demonstrating that the EMT proteins P-cadherin, twist, and HIF-1α are consistently expressed in FOSCC tissue [21].

This report describes the grading and subsequent screening of fourteen feline oronasal SCCs with a known FPV infection status for the expression of epithelial, mesenchymal, and CSC-associated proteins. Tumor grading was carried out according to Anneroth et al. (1987), revealing an invasive disease (grade 3, 4) in all but one case. This finding is not surprising since oral lesions commonly remain unnoticed until they reach an advanced stage characterized by symptoms such as facial deformation, oral blood discharge, weight loss, and dysphagia [2].

In humans, carcinogenic papillomaviruses (high-risk HPVs) are causally associated with HNSCC development in 20 to 50% of cases [1,26,27]. HPV-related lesions are mainly diagnosed in the oropharyngeal region including the base of the tongue and tonsils, whilst HPV-unrelated HNSCCs do not seem to have any predilection site. Importantly, HPV-associated HNSCCs are more sensitive to therapy than their HPV-unrelated counterparts, thus having a better prognosis [27]. Screening of the fourteen feline HNSCCs for FPV DNA revealed the infection in five cases: FPV1 was detected in one mandibular lesion, FPV2 in one linguolabial tumor, and FPV3 in two mandibular and one nasopharyngeal lesion [4]. Given that a total of eight mandibular, three lingual, and two nasopharyngeal SCCs were analyzed, there seems to be no association between FPV infection and tumor localization. This assumption is strengthened by a study on 109 feline SCCs that showed no correlation between FPV DNA detection and the tumor site [28].

Similarly, no correlation between the FPV infection status and the different tumor characteristics determining the stage of disease was observed. This finding was expected based on the reduced sample size. It could be interesting to compare the responsiveness to therapy of FPV-related versus FPV-unrelated HNSCCs to determine whether FPV infection is associated with better prognosis, as observed in HPV-induced HNSCCs [27].

Keratins (KRTs) are a group of intermediate filament proteins of the cytoskeleton. Stratified epithelial KRTs are classified as acidic type I (or low molecular weight—LMW), and basic type 2 (or high molecular weight—HMW) KRTs [29]. IHC screening of feline oronasal SCCs revealed KRT expression in virtually 100% of tumor cells. The staining signal was evenly distributed, delimiting the tumor from adjacent connective tissue.

Vimentin belongs to the group of intermediate filament proteins that maintain the shape, tension, and structural integrity of cells. It is expressed by mesenchymal cells and several immune cell subsets, and is chiefly involved in EMT [30]. In this study, <10 to >50% of tumor cells scored positive for vimentin, suggesting that a certain amount of tumor cells co-expressed KRTs in conjunction with this mesenchymal protein. This assumption was confirmed by double IF staining of tumor sections for KRTs and vimentin demonstrating the intralesional presence of low to high amounts of double-positive cells. In addition, tumor sections scored concurrently positive for the epithelial protein E-cadherin, and the EMT markers N-cadherin and CD146. Co-expression of epithelial and EMT markers agrees with the current concept of partial EMT (pEMT) in solid tumors [16] including HNSCC [17]. Whilst cells undergo full EMT during embryogenesis, EMT of cancer cells remains incomplete, as demonstrated by concurrent expression of epithelial and mesenchymal proteins [18]. From the tumor’s point of view, the pEMT phenotype is extremely advantageous. For example, it confers anoikis resistance on circulating tumor cells (CTCs) and the ability to metastasize more effectively. In addition, it allows collective migration of tumor cells in clusters. This particularly efficient form of invasion is also promoted by tumor-associated fibroblasts (CAFs) that literally pave the way for collective cancer cell migration by creating “microtracks” in the ECM [16,18]. In human HNSCC, pEMT cancer cells were shown to colocalize with CAFs at the tumor front and interact with the latter in a paracrine manner [17].

The transmembrane glycoprotein E-cadherin is a critical determinant of epithelial integrity. It supports cell-to-cell adhesion by linking to the actin cytoskeleton through a tight interplay with catenins, including the prototype member of this family, i.e., β-catenin. [31]. Feline lesions were assessed for β-catenin expression since the interaction of this protein with E-cadherin is often disrupted in tumor cells undergoing EMT, as revealed by translocation of β-catenin to the nucleus, e.g., in human and some equine HNSCCs [31,32,33]. Although all feline lesions exhibited intensive β-catenin staining, the signals were predominantly membranous and occasionally cytoplasmic. Nuclear staining was not observed. These findings agree with our previous observation that β-catenin is normally expressed in equine HNSCCs [22].

COX-2 belongs to a group of enzymes contributing to the conversion of arachidonic acid to prostaglandins (PGs). There is ample evidence of COX-2 deregulating the expression of angiogenic factors, and by this, promoting SCC vascularization and spread [34,35]. PGs converted by COX-2 include PGE2, which has a major role in inflammation and angiogenesis. In human HNSCC, COX-2 contributes to several carcinogenic processes, including tumor cell proliferation, inhibition of apoptosis, local immune suppression, and angiogenesis [36]. Detection of membranous and cytoplasmic COX-2 in all feline HNSCCs suggests that these tumors may respond to therapeutic COX-2 inhibitors such as piroxicam or non-steroidal anti-inflammatory drugs (NSAIDs) [37]. In human HNSCCs, the COX-2 inhibitor celecoxib has shown some effect in oral SCC cell culture [38], and in cats, the safety of piroxicam has been documented [37]. Use of NSAIDs may be of modest benefit. In human HNSCC, only a weak impact on recurrence and overall survival was noted [39].

In humans, CSCs are key determinants of HNSCC progression, metastasis, and MDR [5,11,40]. Although there is only little information on CSCs in non-human SCCs [20,22], we expect this cell subset to likewise promote malignant behavior and therapy resistance in animal cancer patients, including HNSCC-affected cats.

CD44 is usually expressed on the cell surface where it acts as hyaluronan receptor. CD44 was the first cluster of differentiation molecule to be identified as CSC marker in human cancer disease, including HNSCC [5,11,14,40]. Interestingly, the CD44 gene contains a central region of nine exons that can be alternatively spliced, giving rise to CD44 variant isoforms (CD44v) with additional pro-tumor functions [41]. CD271, also known as low-affinity nerve growth factor receptor (LNGFR), or neutrophin receptor (p75NTR), belongs to the tumor necrosis factor family [42,43]. CD271 is considered the most efficient marker of human bone marrow mesenchymal stem cells [44]. In addition, it has gained in significance as a CSC marker [40,45] because of the growing evidence that CSCs constitute a CD271^+^ subset within the CD44^+^ tumor cell compartment [14]. The feline lesions were assessed for the presence of CSCs by single and combined CD44/CD271 immunostaining. This approach helped distinguish between CD271-positive cancer cells and lymphocytes that also express this protein [45], and allowed for the detection of CD44/CD271 double-positive cells that likely represent CSCs. Importantly, single CD44 staining revealed <10 to >50% positive cells in all but one lesion, and in most cases, the signal intensity was particularly pronounced within infiltrative tumor fronts. Moreover, considerable numbers of cells expressed CD44 in conjunction with CD271 in all but one lesion. This finding indicates that high-grade feline oronasal SCC is associated with the transformation of tumor cells from an epithelial to a cancer stem cell-like phenotype.

Feline HNSCC CSCs may represent an interesting therapeutic target. For example, recent in vitro and murine data point to the polyether antibiotic salinomycin (SAL) selectively eradicating multidrug-resistant cancer cells, notably CSCs, by interference with e.g., Akt, Wnt/ β-catenin, Hedgehog, and Notch cancer progression pathways [46,47,48]. This interference also results in SAL potentially acting as an inhibitor of EMT processes. In addition, SAL can inhibit the overexpression P-glycoprotein (P-gp) in cancer cells that act as a drug efflux pump and thus promotes multidrug resistance (MDR). Importantly, SAL-mediated inhibition of multidrug-resistant tumor cells seems to restore the overall sensitivity of tumors to various approved treatments [49]. Provided that down-stream analyses can unequivocally demonstrate the presence and pathobiological role of feline CSCs in the near future, the authors’ current focus on the establishment of a contrast agent-coupled SAL as an innovative theranostic in the management of canine melanoma could be extended to feline HNSCC.

It is well established that CD44 and its splicing variants have a crucial role in the maintenance and function of CSCs in many human cancer diseases [41]. In human HNSCC, there is evidence that CD44v3 promotes tumor cell proliferation, migration, and cisplatin resistance in vitro and in vivo [50,51,52]. In feline HNSCC, the existence and possible pathobiological role of CD44 isoforms are still unexplored. Research efforts should be directed to this field, all the more since CD44 and its isoforms could emerge as valuable prognostic markers and therapeutic targets.

## 5. Conclusions

The herein presented work is innovative as it provides unprecedented insights into cancer cell plasticity in feline HNSCC. However, the study has limitations with respect to the relatively small sample size. This had to be considered when interpreting results. Obtained findings indicate that feline HNSCCs closely resemble their human counterparts in that cancer cells show a similar ability to switch from an epithelial to a pEMT phenotype and also acquire a protein expression pattern complying with the CSC phenotype. The demonstrated plasticity of feline HNSCC cells likely contributes to the malignancy of the disease, as similarly described for human HNSCC, and more generally for solid cancers in the human species. Future work will aim at confirming this assumption by in-depth studies in a larger number of FPV-positive and -negative tumor samples. Special emphasis will be put on the further characterization of CSCs as well as the identification and role of CD44 isoforms in feline HNSCCs to pave the way towards the improvement of feline HNSCC management.

## Figures and Tables

**Table 1:** Patient and sample specifications including feline papillomavirus PCR results.

No.	Code	Breed	Age in Years	Sex	Tumor Localization	FPV Infection Status
1	CAL	Norwegian Forest	14	fs	mandibular	FPV3
2	JAC	ESH	14	m	mandibular	FPV1
5	MAX	ESH	8	mc	mandibular	FPV3
8	MIM	ESH	12	fs	lingual	negative
6	MIN	ESH	13	fs	mandibular	negative
10	MMI	ESH	15	fs	mandibular	negative
7	NEL	Persian	12	fs	lingual	negative
12	NIK	ESH	3	mc	lingual, labial	FPV2
9	PAG	Persian	14	fs	oral	negative
14	PUP	ESH	14	fs	nasopharyngeal, orbital	negative
3	SHA	Abyssinian	12	mc	mandibular	negative
4	SNO	ESH	15	fs	mandibular	negative
11	SUR	ESH	11	fs	nasopharyngeal	FPV3
13	SUS	ESH	14	fs	mandibular	negative

No.—number; ESH—European Shorthair; fs—female spayed; m—male; mc—male castrated. FPV1, 2, 3—feline papillomavirus type 1, 2, or 3.

**Table 2:** Histological grading of feline HNSCCs.

Code	KRT	NPM	Mitotic Cells/HPF	Invasion Pattern	Invasion Stage	LPI	Total Score	Grade
CAL *	1	2	1	2	4	4	14	2
JAC *	2	3	4	4	4	4	21	4
MAX	4	2	4	3	3	2	18	3
MIM	4	3	4	4	4	3	22	4
MIN	3	3	4	3	4	1	18	3
MMI	4	2	ne *	4	4	4	19+	3–4
NEL	4	3	4	4	4	2	21	4
NIK *	4	3	4	4	4	2	21	4
PAG	3	2	ne *	4	3	4	17+	3
PUP	3	2	1	4	4	4	18	3
SHA*	4	4	4	2	4	4	22	4
SNO	3	3	ne *	4	4	3	18+	3
SUR *	3	3	4	2	4	3	19	3
SUS	4	4	4	4	4	3	23	4

*: FPV DNA detected in tumor material; KRT—keratinization; NPM—nuclear polymorphism; HPF—high-power field; LPI—lymphoplasmacytic infiltration; ne—non-evaluable; * tumor did not extend towards the margins of 10 high-power fields. Grade 1 = 5–10 points; grade 2 = 11–15 points; grade 3 = 16–20 points; grade 4 > 20 points.

## Data Availability

All relevant data are presented in this article.

## Supplementary Materials

The following supporting information can be downloaded at: https://www.mdpi.com/article/10.3390/pathogens12111288/s1, Table S1: Antibody specifications for IHC single staining and IF double staining.

## References

[1] Dayyani F., Etzel C.J., Liu M., Ho C.H., Lippman S.M., Tsao A.S. (2010). Meta-analysis of the impact of human papillomavirus (HPV) on cancer risk and overall survival in head and neck squamous cell carcinomas (HNSCC). Head Neck Oncol..

[2] Withrow S.J., Vail D.M., Page R.L. (2013). Squamous cell carcinoma. Withrow & MacEwen’s Small Animal Clinical Oncology.

[3] Sequeira I., Pires M.D.A., Leitao J., Henriques J., Viegas C., Requicha J. (2022). Feline Oral Squamous Cell Carcinoma: A Critical Review of Etiologic Factors. Vet. Sci..

[4] Altamura G., Cuccaro B., Eleni C., Strohmayer C., Brandt S., Borzacchiello G. (2022). Investigation of multiple Felis catus papillomavirus types (-1/-2/-3/-4/-5/-6) DNAs in feline oral squamous cell carcinoma: A multicentric study. J. Vet. Med. Sci..

[5] Salem A., Salo T. (2023). Identity matters: Cancer stem cells and tumour plasticity in head and neck squamous cell carcinoma. Expert Rev. Mol. Med..

[6] Hanahan D., Weinberg R.A. (2011). Hallmarks of cancer: The next generation. Cell.

[7] Thiery J.P., Acloque H., Huang R.Y., Nieto M.A. (2009). Epithelial-mesenchymal transitions in development and disease. Cell.

[8] Yang J., Weinberg R.A. (2008). Epithelial-mesenchymal transition: At the crossroads of development and tumor metastasis. Dev. Cell.

[9] Gonzalez-Gonzalez R., Ortiz-Sarabia G., Molina-Frechero N., Salas-Pacheco J.M., Salas-Pacheco S.M., Lavalle-Carrasco J., Lopez-Verdin S., Tremillo-Maldonado O., Bologna-Molina R. (2021). Epithelial-Mesenchymal Transition Associated with Head and Neck Squamous Cell Carcinomas: A Review. Cancers.

[10] Hujanen R., Almahmoudi R., Salo T., Salem A. (2021). Comparative Analysis of Vascular Mimicry in Head and Neck Squamous Cell Carcinoma: In Vitro and In Vivo Approaches. Cancers.

[11] Oshimori N. (2020). Cancer stem cells and their niche in the progression of squamous cell carcinoma. Cancer Sci..

[12] Prince M.E., Sivanandan R., Kaczorowski A., Wolf G.T., Kaplan M.J., Dalerba P., Weissman I.L., Clarke M.F., Ailles L.E. (2007). Identification of a subpopulation of cells with cancer stem cell properties in head and neck squamous cell carcinoma. Proc. Natl. Acad. Sci. USA.

[13] Xiao M., Liu L., Zhang S., Yang X., Wang Y. (2018). Cancer stem cell biomarkers for head and neck squamous cell carcinoma: A bioinformatic analysis. Oncol. Rep..

[14] Yu S.S., Cirillo N. (2020). The molecular markers of cancer stem cells in head and neck tumors. J. Cell Physiol..

[15] Liao C., Wang Q., An J., Long Q., Wang H., Xiang M., Xiang M., Zhao Y., Liu Y., Liu J. (2021). Partial EMT in Squamous Cell Carcinoma: A Snapshot. Int. J. Biol. Sci..

[16] Saitoh M. (2018). Involvement of partial EMT in cancer progression. J. Biochem..

[17] Kisoda S., Mouri Y., Kitamura N., Yamamoto T., Miyoshi K., Kudo Y. (2022). The role of partial-EMT in the progression of head and neck squamous cell carcinoma. J. Oral. Biosci..

[18] Shibue T., Weinberg R.A. (2017). EMT, CSCs, and drug resistance: The mechanistic link and clinical implications. Nat. Rev. Clin. Oncol..

[19] Okuyama K., Suzuki K., Yanamoto S. (2023). Relationship between Tumor Budding and Partial Epithelial-Mesenchymal Transition in Head and Neck Cancer. Cancers.

[20] Pang L.Y., Bergkvist G.T., Cervantes-Arias A., Yool D.A., Muirhead R., Argyle D.J. (2012). Identification of tumour initiating cells in feline head and neck squamous cell carcinoma and evidence for gefitinib induced epithelial to mesenchymal transition. Vet. J..

[21] Harris K., Gelberg H.B., Kiupel M., Helfand S.C. (2019). Immunohistochemical Features of Epithelial-Mesenchymal Transition in Feline Oral Squamous Cell Carcinoma. Vet. Pathol..

[22] Strohmayer C., Klang A., Kummer S., Walter I., Jindra C., Weissenbacher-Lang C., Redmer T., Kneissl S., Brandt S. (2022). Tumor Cell Plasticity in Equine Papillomavirus-Positive Versus-Negative Squamous Cell Carcinoma of the Head and Neck. Pathogens.

[23] Anneroth G., Batsakis J., Luna M. (1987). Review of the literature and a recommended system of malignancy grading in oral squamous cell carcinomas. Scand. J. Dent. Res..

[24] Wang Z., Yan X. (2013). CD146, a multi-functional molecule beyond adhesion. Cancer Lett..

[25] Aiello N.M., Kang Y. (2019). Context-dependent EMT programs in cancer metastasis. J. Exp. Med..

[26] Feller L., Wood N.H., Khammissa R.A., Lemmer J. (2010). Human papillomavirus-mediated carcinogenesis and HPV-associated oral and oropharyngeal squamous cell carcinoma. Part 2: Human papillomavirus associated oral and oropharyngeal squamous cell carcinoma. Head Face Med..

[27] Fleming J.C., Woo J., Moutasim K., Mellone M., Frampton S.J., Mead A., Ahmed W., Wood O., Robinson H., Ward M. (2019). HPV, tumour metabolism and novel target identification in head and neck squamous cell carcinoma. Br. J. Cancer.

[28] Yamashita-Kawanishi N., Chang C.Y., Chambers J.K., Uchida K., Sugiura K., Kukimoto I., Chang H.W., Haga T. (2021). Comparison of prevalence of Felis catus papillomavirus type 2 in squamous cell carcinomas in cats between Taiwan and Japan. J. Vet. Med. Sci..

[29] Moll R., Franke W.W., Schiller D.L., Geiger B., Krepler R. (1982). The catalog of human cytokeratins: Patterns of expression in normal epithelia, tumors and cultured cells. Cell.

[30] Arrindell J., Desnues B. (2023). Vimentin: From a cytoskeletal protein to a critical modulator of immune response and a target for infection. Front. Immunol..

[31] Kudo Y., Kitajima S., Ogawa I., Hiraoka M., Sargolzaei S., Keikhaee M.R., Sato S., Miyauchi M., Takata T. (2004). Invasion and metastasis of oral cancer cells require methylation of E-cadherin and/or degradation of membranous beta-catenin. Clin. Cancer Res..

[32] Armando F., Godizzi F., Razzuoli E., Leonardi F., Angelone M., Corradi A., Meloni D., Ferrari L., Passeri B. (2020). Epithelial to Mesenchymal Transition (EMT) in a Laryngeal Squamous Cell Carcinoma of a Horse: Future Perspectives. Animals.

[33] Stenner M., Yosef B., Huebbers C.U., Preuss S.F., Dienes H.P., Speel E.J., Odenthal M., Klussmann J.P. (2011). Nuclear translocation of beta-catenin and decreased expression of epithelial cadherin in human papillomavirus-positive tonsillar cancer: An early event in human papillomavirus-related tumour progression?. Histopathology.

[34] Millanta F., Andreani G., Rocchigiani G., Lorenzi D., Poli A. (2016). Correlation Between Cyclo-oxygenase-2 and Vascular Endothelial Growth Factor Expression in Canine and Feline Squamous Cell Carcinomas. J. Comp. Pathol..

[35] Toomey D., Conroy H., Jarnicki A.G., Higgins S.C., Sutton C., Mills K.H. (2008). Therapeutic vaccination with dendritic cells pulsed with tumor-derived Hsp70 and a COX-2 inhibitor induces protective immunity against B16 melanoma. Vaccine.

[36] Frejborg E., Salo T., Salem A. (2020). Role of Cyclooxygenase-2 in Head and Neck Tumorigenesis. Int. J. Mol. Sci..

[37] DiBernardi L., Dore M., Davis J.A., Owens J.G., Mohammed S.I., Guptill C.F., Knapp D.W. (2007). Study of feline oral squamous cell carcinoma: Potential target for cyclooxygenase inhibitor treatment. Prostaglandins Leukot. Essent. Fatty Acids.

[38] Janakiraman H., House R.P., Talwar S., Courtney S.M., Hazard E.S., Hardiman G., Mehrotra S., Howe P.H., Gangaraju V., Palanisamy V. (2017). Repression of caspase-3 and RNA-binding protein HuR cleavage by cyclooxygenase-2 promotes drug resistance in oral squamous cell carcinoma. Oncogene.

[39] Saka-Herran C., Jane-Salas E., Estrugo-Devesa A., Lopez-Lopez J. (2021). Head and neck cancer and non-steroidal anti-inflammatory drugs: Systematic review and meta-analysis. Head. Neck.

[40] Sadasivam S., Subramanian R. (2020). A perspective on challenges and opportunities in characterizing oral cancer stem cells. Front. Biosci. (Landmark Ed).

[41] Chen C., Zhao S., Karnad A., Freeman J.W. (2018). The biology and role of CD44 in cancer progression: Therapeutic implications. J. Hematol. Oncol..

[42] Chesa P.G., Rettig W.J., Thomson T.M., Old L.J., Melamed M.R. (1988). Immunohistochemical analysis of nerve growth factor receptor expression in normal and malignant human tissues. J. Histochem. Cytochem..

[43] Thomson T.M., Rettig W.J., Chesa P.G., Green S.H., Mena A.C., Old L.J. (1988). Expression of human nerve growth factor receptor on cells derived from all three germ layers. Exp. Cell Res..

[44] Alvarez-Viejo M., Menendez-Menendez Y., Otero-Hernandez J. (2015). CD271 as a marker to identify mesenchymal stem cells from diverse sources before culture. World J. Stem Cells.

[45] Vidal A., Redmer T. (2020). Decoding the Role of CD271 in Melanoma. Cancers.

[46] Antoszczak M., Huczynski A. (2019). Salinomycin and its derivatives - A new class of multiple-targeted “magic bullets”. Eur. J. Med. Chem..

[47] Liu Q., Sun J., Luo Q., Ju Y., Song G. (2021). Salinomycin Suppresses Tumorigenicity of Liver Cancer Stem Cells and Wnt/Beta-catenin Signaling. Curr. Stem. Cell Res. Ther..

[48] Mai T.T., Hamai A., Hienzsch A., Caneque T., Muller S., Wicinski J., Cabaud O., Leroy C., David A., Acevedo V. (2017). Salinomycin kills cancer stem cells by sequestering iron in lysosomes. Nat. Chem..

[49] Dewangan J., Srivastava S., Rath S.K. (2017). Salinomycin: A new paradigm in cancer therapy. Tumour Biol..

[50] Bourguignon L.Y., Wong G., Earle C., Chen L. (2012). Hyaluronan-CD44v3 interaction with Oct4-Sox2-Nanog promotes miR-302 expression leading to self-renewal, clonal formation, and cisplatin resistance in cancer stem cells from head and neck squamous cell carcinoma. J. Biol. Chem..

[51] Reategui E.P., de Mayolo A.A., Das P.M., Astor F.C., Singal R., Hamilton K.L., Goodwin W.J., Carraway K.L., Franzmann E.J. (2006). Characterization of CD44v3-containing isoforms in head and neck cancer. Cancer Biol. Ther..

[52] Wang S.J., Wreesmann V.B., Bourguignon L.Y. (2007). Association of CD44 V3-containing isoforms with tumor cell growth, migration, matrix metalloproteinase expression, and lymph node metastasis in head and neck cancer. Head Neck.

