# Supplementary material for: Feline SCCs of the Head and Neck Display Partial Epithelial-Mesenchymal Transition and Harbor Stem Cell-like Cancer Cells
Source: Pathogens. 2023 Oct 27;12(11):1288. doi: 10.3390/pathogens12111288 (PMC10674711; doi:10.3390/pathogens12111288)
Supplement: Supplementary file 1 [file pathogens-12-01288-s001.zip › pathogens-2675709-supplementary.pdf]

Supplementary File S1

Table S1: Antibody specifications for IHC single staining and IF double staining

**IHC (SINGLE STAINING)**						
**Primary Antibodies**						
**Host**	**Type**	**Clone**	**Target Protein**	**Provider**	**Dilution**	**HIER**
Mouse	Monoclonal	AE1	LMW keratins	Cell Marque, Sigma-Aldrich, Vienna, Austria	1:650	HIER, pH 9
Mouse	Monoclonal	AE3	HMW keratins	Cell Marque, Sigma-Aldrich, Vienna, Austria	1:650	HIER, pH 9
Mouse	Monoclonal	9G2	beta-Catenin	Acris Antibodies, Herford, Germany	1:500	HIER, pH 9
Mouse	Monoclonal	V9	Vimentin	Dako, Hamburg, Germany	1:500	HIER, pH 6
Rabbit	Recombinant	EPR3208	CD146	Abcam, Cambridge, UK	1:500	HIER, pH 6
Rabbit	Monoclonal	SP21	COX2	Thermo Fisher Scientific, Vienna, Austria	1:400	HIER, pH 6
Rabbit	Monoclonal	D4B3	CD271 (p75NTR)	Cell Signaling Technology, Frankfurt, Germany	1:1000	HIER, pH 9
Rat	Monoclonal	IM7	CD44	Santa Cruz Biotechnology, Dallas, Texas, USA	1:200	HIER, pH 6

**Secondary Antibodies**	
**Description**	**Provider**
Goat anti-mouse Ab-Poly HRP, ready to use	BrightVision, ImmunoLogic, Duiven, The Netherlands
Goat anti-rabbit Ab-Poly HRP, ready to use	BrightVision, ImmunoLogic, Duiven, The Netherlands
Goat anti-rat Ab-HRP, 1:500	Abcam, Cambridge, UK

**IF (DOUBLE STAINING)**						
**Primary Antibodies**						
**Host**	**Type**	**Clone**	**Target**	**Provider**	**Dilution**	**Pretreatment**
Rat	Monoclonal	IM7	CD44	Santa Cruz Biotechnology, Dallas, Texas, USA	1:500	HIER, pH 9
Rabbit	Monoclonal	D4B3	CD271 ($p75^{NTR}$)	Cell Signaling Technology, Frankfurt, Germany	1:250	HIER, pH 9
Mouse	Monoclonal	AE1	LMW keratins	Cell Marque, Sigma-Aldrich, Vienna, Austria	1:400	HIER, pH 9
Mouse	Monoclonal	AE3	HMW keratins	Cell Marque, Sigma-Aldrich, Vienna, Austria	1:400	HIER, pH 9
Rabbit	Polyclonal	-	Vimentin	Merck, Sigma-Aldrich, Darmstadt, Germany	1:500	HIER, pH 9

**Secondary Antibodies**	
**Description**	**Provider**
Goat anti-rat Ab Alexa Fluor 488, 1:500	Invitrogen, Thermo Fisher Scientific, Vienna, Austria

Goat anti-rabbit Ab-Alexa Fluor 568, 1:1500	Invitrogen, Thermo Fisher Scientific, Vienna, Austria
Donkey anti-mouse Ab-Alexa Fluor 488, 1:500	Jackson ImmunoResearch Europe Ltd, Ely, UK
Donkey anti-rabbit Ab-Alexa Fluor 568, 1:400	Invitrogen, Thermo Fisher Scientific, Vienna, Austria

IF (TYRAMIDE AMPLIFICATION)

Primary Abs

**Host**	**Type**	**Clone**	**Target**	**Provider**	**Dilution**	**Pretreatment**
Mouse	Monoclonal	4A2C7	E-Cadherin (CDH1)	Invitrogen, Thermo Fisher Scientific, Vienna, Austria	1:500	HIER, pH 9
Mouse	Monoclonal	13A9	N-Cadherin (CDH2)	Santa Cruz Biotechnology, Dallas, Texas, USA	1:400	HIER, pH 9

Secondary Antibodies / Development

**Description**	**Provider**
Poly HRP anti-mouse Ab, ready to use	BrightVision, Immunologic, Duiven, The Netherlands
Alexa Fluor 488 Tyramide Reagent	Invitrogen, Thermo Fisher Scientific, Vienna, Austria
Alexa Fluor 568 Tyramide Reagent	Invitrogen, Thermo Fisher Scientific, Vienna, Austria

Abbreviations: HIER = heat induced epitope retrieval; Ab = antibodies.
